# Intramedullary knee spacer in 2-stage revision knee surgery with segmental bone loss: A technical note involving 6 cases

**DOI:** 10.3109/17453674.2012.694778

**Published:** 2012-06-04

**Authors:** Henrik M Schrøder, Michael M Petersen

**Affiliations:** Department of Orthopaedic Surgery U-2162, Rigshospitalet, University Hospital of Copenhagen, Denmark

In 2-stage revision knee surgery ending with a tumor prosthesis, the bone loss is so large that in the interim period the patient can be treated with (1) rest in bed with or without skeletal traction, (2) external fixation, or (3) a custom-made spacer. Apart from the technical challenge of eradicating these infections, the stabilization of the knee and preservation of leg length until reimplantation can be a problem. We present our early experience with a new type of knee spacer for patients with very large bone loss.

## Surgical technique

**Figure 1.**
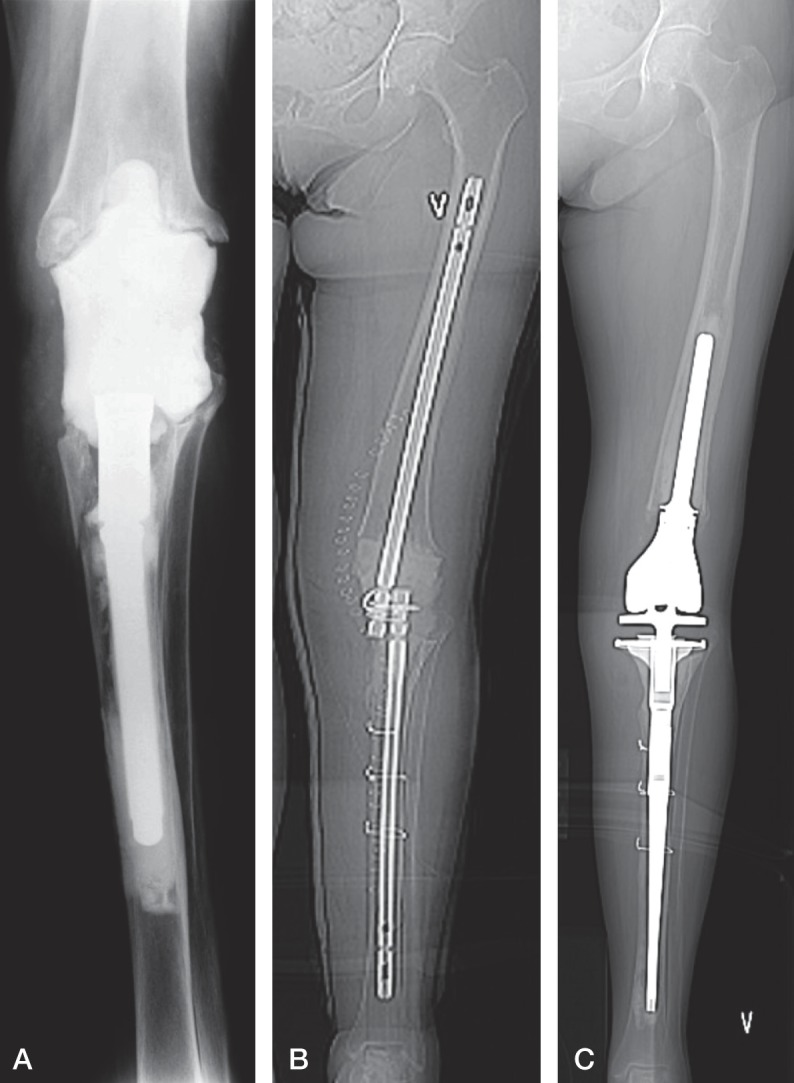
A 79-year-old woman with candida ostitis, who had the least bone loss on the femoral side, and the most bone loss on the tibial side. A. Status at referral with a cement spacer and the tibial stem still well-fixed to the bone. B. After revision, including removal of the tibial stem and insertion of an intramedullary spacer made by 2 retrograde nails connected with wires plus bone cement. C. X-ray after removal of the spacer and insertion of a tumor prosthesis (C).

**Figure 2.**
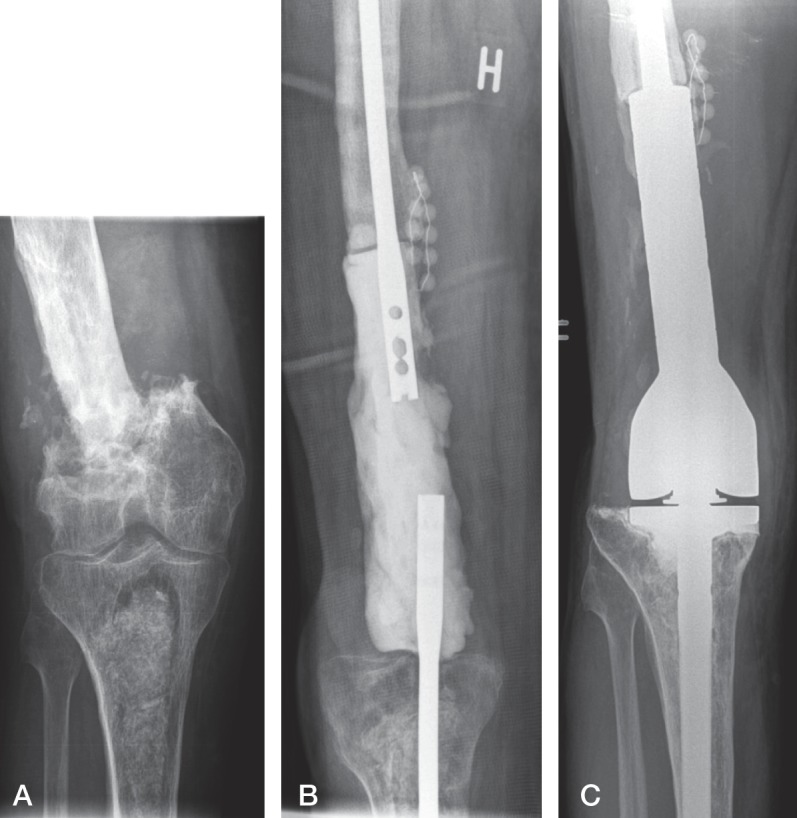
A 37-year-old man with an infected pseudartrosis. A. Preoperatively; pseudarthrosis of the distal femur. B. Spacer inserted after revision, with resection of all infected and dead bone. C. After stage 2 (one year postoperatively), with tumor prosthesis inserted.

A thigh tourniquet was used if possible. An anterior incision extending proximally was done, extending the old incision. After thorough debridement with removal of hardware, all infected and/or necrotic bone was resected. Remaining tibial and femoral canals were reamed and sized with pulse lavage irrigation before and after reaming. Retrograde femoral IM nails (AO) were inserted by press-fit in the full lengths of the femoral and tibial canals under fluoroscopic guidance. In the first 2 cases, 1 of the nails was locked; in the last 4 cases, no locking screws were used. Thereafter, the assistant pulled the leg into maximum possible length while the free ends of the nails were cemented together using 3–5 portions of bone cement as a spacer between the femur and tibia. In 4 cases, the nails inserted were so long that the free ends could be connected with 1 or 2 screws through the locking holes before cementing. After cementing, all metal between the bony ends of the femur and tibia was covered with cement, but no cement was put into the canals. The spacer was thus made of 2 retrograde intramedullary nails, preferably connected with 1 or 2 screws or wires, and the “free ends” of the nails were embedded in cement (Refobacin Revision; Biomet) (Figures 1 and 2), which contained clindamicin and gentamicin. Amphotericin B was added in a patient with candida infection. The patients were mobilized with 2 crutches and toe-touch weight bearing. A softcast cylinder was optional during mobilization.

## Patients

Between 2006 and 2008, we operated 6 selected patients with this new spacer technique. 5 of the patients had an infected revision knee arthroplasty (Figure 1) and 1 had a posttrauma-infected distal femoral pseudarthrosis (Figure 2). The mean number of previous operations was 7 (2–11) and mean age at the spacer operation was 63 (37–79) years. In all 6 patients femoral bone loss was extensive, ranging from supracondylarly to the middle of the femur, thus exceeding type III in the AORI classification (Engh and Ammeen 1998). The tibial bone loss was type II (2) or type III (4). In all patients, the extensor mechanism was intact, and despite open drainage in 3 patients, no plastic surgery was needed. Four patients had been recommended amputation above the knee before referral.

The microbiology was mixed in 4 patients (4 *Staphyloccocus epidermidis,* 3 *Enterococcus faecalis,* 2 *Staphyloccocus aureus,* 1 *Enterobacter cloacae,* and 1 *Escherichia coli*), and single in 2 patients (1 *Staphyloccocus epidermidis,* 1 *Candida parapsilosis*). The first stage was followed by treatment with systemic antibiotics (with guidance from a clinical microbiologist) until clinical eradication, and normalized inflammatory markers. Then the second stage with implantation of a tumor prosthesis was done.

The mean period with IM spacer was 3 (1.5–6.5) months, and the leg length shortening was 3.5 (0–10) cm preoperatively and 2 (0–3.5) cm after insertion of the tumor prosthesis. Mean ROM was 27 (10–50) degrees preoperatively and 58 (35–105) degrees after stage 2.

1 spacer fractured after 1 week. The patient was reoperated, but the spacer fractured again and the knee was left with considerable shortening in a plaster for 2.5 months before successful implantation of a tumor prosthesis. This heavy male patient did not respect our advice not to bear full weight and for a period was considered a candidate for amputation. Apart from the patient with a fractured spacer, no reoperations were needed before reimplantation; 5 patients had a GMRS tumor prosthesis (Stryker) and 1 had a MEGA-C tumor prosthesis (Valdemar Link).

2 patients died: 1 died 5 weeks postoperatively and 1 died 5 years postoperatively. Both were free of infection and died because of cardiac disease. 4 patients are still alive (as of December 2011) and free of infection. 2 patients have been reoperated after reimplantation, both after about 1 year (1 aseptic loosening of the femoral stem and 1 breakage of a femoral stem connection).

## Discussion

We consider the present spacer technique to be safe, as all infections were cured. All hardware implanted during the first stage is usually covered with antibiotic-loaded cement, both in non-tumor cases (Antoci et al. 2009, Incavo et al. 2009, Rao et et al. 2009) and tumor cases (Grimer et al. 2002, Manoso eal. 2006), so our technique is truly new and controversial. The fact that all infections were cured despite the use of uncoated IM nails can be attributed to aggressive debridement at stage 1 with resection of all bone tissue that was not considered vital and free of infection, plus full cement coating of the nails in the primarily infected area. It is noteworthy that no further surgery for infection was needed after this first stage.

The small amount of literature that is available does not favor 1-stage revisions in complex cases with substantial bone loss and frequent fistula/skin problems (Ramappa et al. 2010). The size of the bone loss in our patients did not permit the implantation of unconstrained or semi-constrained spacers, which have been described by others as being suited for large bone loss (Incavo et al. 2009, MacAvoy and Ries 2005), and perhaps the soft tissues even profited from the immobilization. Despite the large number of previous operations and a mean of 3 months with a stiff knee, the movement achieved after stage 2 was acceptable.

The usual spacer technique in very large bone loss is insertion of 1 antibiotic-covered rod (Grimer et al. 2002, Antoci et al. 2009, Rao et al. 2009) or a prosthesis (Sherman et al. 2008). Our IM spacer is probably even more mechanically stable and is technically easy, both to perform and—also important—to remove without further loss of bone during stage 2. It is also applicable in infected tumor cases, when tumor itself is not an issue.

Our method ensures preservation of leg length and makes ambulation and nursing between the 2 stages very easy compared to the traditional 2–3 months of skeletal traction in bed. External fixation (Berend and Lombardi 2009) will also favor ambulation, but introduces an additional risk of infection and weakens the rest of the femur where the tumor prosthesis will be implanted.

Our technique requires enough bone, on both the femoral and tibial side, to first insert an intramedullary nail in press-fit, and secondly to implant a prosthetic stem. A remaining proximal femoral bony canal of less than about 15 cm probably excludes our technique, and skeletal traction in a bed and subsequent total femoral replacement will be the choice (Berend et al. 2004). Tibial bone loss of that size will probably lead to amputation, but a modular cemented nail (Rao et al. 2009)—or in rare cases a tumor prosthesis (Russell et al. 2008)—might be an alternative. Amputation is sometimes considered in patients with persistent periprosthetic or posttraumatic infection of the knee despite several operations, and is indeed an effective operation regarding cure of infection. However, mobility decreases, and a tumor prosthesis actually gives good functional results (Berend and Lombardi 2010) and even seems to be cost effective compared to amputation (Grimer et al. 1997).

On the basis of our early experience, we recommend that the 2 nails be inserted in press-fit without the use of locking screws. The connection of the free ends of the nails can be reinforced with 1 or 2 screws before applying cement, but can also be connected with wires. The trick is to secure the leg length before cementing.

In conclusion, the present intramedullary knee spacer appears to be an effective, safe, and patient-friendly method at the first stage of 2-stage revisions, where bone loss is so large that a tumor prosthesis will be needed at stage 2.
